# Species Assembly of Highland Anuran Communities in Equatorial Africa (Virunga Massif): Soundscape, Acoustic Niches, and Partitioning

**DOI:** 10.3390/ani14162360

**Published:** 2024-08-15

**Authors:** Ulrich Sinsch, Deogratias Tuyisingize, Jonas Maximilian Dehling, Yntze van der Hoek

**Affiliations:** 1 Institute of Integrated Sciences, Department of Biology, University of Koblenz, D-56070 Koblenz, Germany; dehling@uni-koblenz.de; 2 Dian Fossey Gorilla Fund, Ellen DeGeneres Campus, Kinigi P.O. Box 105, Rwanda; dtuyisingize@gorillafund.org (D.T.); yvanderhoek@gorillafund.org (Y.v.d.H.)

**Keywords:** soundscape, passive automated monitoring, advertisement call, calling activity, acoustic niche hypothesis, stochastic niche theory

## Abstract

**Simple Summary:**

Male anurans attract conspecific females for reproduction by giving advertisement calls. As calling implies high energetic costs, theory predicts that in mixed-species assemblages call features differ among species to reduce acoustic interference. Each species occupies a distinct acoustic niche in the soundscape. In equatorial wetlands water bodies for reproduction are available all over the year, therefore, we expect that diel and seasonal partitioning of acoustic space should be less important than differences in call structure to reduce competition and niche overlap. Additionally, acoustic communities should be species-saturated. We tested these predictions in four communities at 2546–3188 m a.s.l. in the Volcanoes National Park, Rwanda. Using automated recording devices from September 2019 to March 2020, we obtained an extensive dataset on call activity. The communities included 4–6 species, depending on the wetland structure, with a common stock of three species (*Hyperolius castaneus*, *H. glandicolor*, *Leptopelis kivuensis*). Independent of elevation, niche breadth for call features was similar and overlap reduced by partitioning the frequency range used. The diel and seasonal niche breadth of specific call activity varied according to the local temperature regime at different altitudes. We conclude that stochastic niche theory describes accurately the processes shaping equatorial highland communities.

**Abstract:**

The soundscape is a complex arrangement of sounds originating from animals and the environment. It is considered a reliable proxy for ecosystem niche structure at the community level. Acoustic communities of anuran species include advertising males, which compete in acoustic space for conspecific females. Stochastic niche theory predicts that all local niches are occupied, and the acoustic community is species-saturated. Acoustic niches, which include the spectral and temporal call structure and diel and seasonal patterns of call activity, are of similar breadth with small overlap. We tested these predictions in four communities inhabiting pristine wetlands at 2546–3188 m a.s.l. in the Volcanoes National Park, Rwanda. We sampled 74 days of hourly 5 min recordings of the local soundscape (September 2019–March 2020) using passive automated monitoring devices (Songmeter SM4). We identified species based on the advertisement call features and measured call activity as calls per minute. The communities included 4–6, species depending on wetland structure, with a shared stock of three species (*Hyperolius castaneus*, *H. glandicolor*, *Leptopelis kivuensis*). Independent of elevation, niche breadth for call features was similar among species and overlap reduced by partitioning the frequency range used. The diel and seasonal niche breadth of specific call activity varied according to the local temperature regime at different altitudes representing the variable part of the acoustic niche. We conclude that communities are indeed species-saturated and acoustic niches differ primarily by the fixed call features remaining locally adaptable by the modulation of the call activity pattern, corroborating the predictions of the stochastic niche theory.

## 1. Introduction

Soundscape ecology is an eco-ethological research discipline that studies the acoustic relationships between living organisms and their environment [1]. The soundscape is a complex arrangement of sounds from multiple sources including biophony, geophony, and anthrophony that creates acoustical patterns in space and time [2]. For example, forests, grasslands, and wetlands support a wide array of biophonic sounds produced by mammals, birds, amphibians, and insects [3]. Eco-acoustic approaches rely on the assumption that animal and environmental sounds are a reliable proxy for the ecosystem complexity up to the community level [4,5,6,7]. Thus, an acoustic community is defined as an aggregation of species that produce sound and its signature results from the propagation of characteristic calls varying in signal amplitude and frequency (call structure), in time (diel and seasonal) and in space (microhabitat) [5]. When calls overlap in frequency and time, acoustic interference and signal masking may occur [8]. Consequently, Krause developed the acoustic niche hypothesis (ANH), which predicts that signaling behavior has evolved to partition acoustic space and to minimize overlap with other sound producing individuals through selection on call structure, diel and seasonal timing of calling, and the site of call propagation [9,10]. ANH is an extension of the Hutchinson niche theory [11], considering acoustic space as a resource that organisms may compete for and that can be partitioned to reduce competition. The classical tradeoff-based niche theory predicts that the number of species forming a community have similar fundamental niches, which are evolutionary and ecologically constrained due to the similar use of resources, whereas the neutral niche theory assumes that species are ecologically equivalent in their responses to all constraints and thus have no interspecific tradeoffs (see review in [12]). Tradeoff-based theories do not predict a limit to diversity, whereas neutral theories assume a threshold diversity representing a balance between speciation and stochastic extinction independent of species traits. The stochastic niche theory complements competitive tradeoff theory with stochastic processes underlying neutral theories [12]. Three predictions about community structure follow: (1) stochastic niche assembly creates communities in which species occupy about similar niche breadth; (2) if local resource levels do not change significantly in time, the established species of a community do not leave resource slots unused for invading species; (3) species-saturated communities created by stochastic competitive assembly have generally a low invasibility.

Empiric studies testing the ANH in anuran and bird communities are generally concerned with the partitioning of single niche dimensions without focusing on community assembly [4,8,13,14,15,16,17]. As predicted by tradeoff-based niche theories, partitioning concerned mostly spectral and temporal call features but also the diel and seasonal timing of calling activity and the spatial segregation of call sites, while, contrary to predictions, vocal synchronization occurred in some bird communities [15]. Usually, the calling activities of anuran amphibians take place in low-noise environments, but biophonic (syntopic birds, mammals, insects), geophonic (wind, rain), and anthrophonic background noise (traffic noise) may interfere with the acoustic communication of a species [18,19,20]. The main response of anurans is avoiding periods of loud environmental noise, as well as stopping or reducing the number of calls [21,22]. Species with low dominant call frequencies tended more to gap-calling behavior than those with high frequencies [19]. The sensitivity of anuran calling activity to masking noise is because the production of advertisement calls includes high energetic costs [23]. Thus, anurans are expected to choose an appropriate activity time when they can call with less energy by minimizing the influences of abiotic factors and biotic factors [19,22,24,25,26].

Pristine habitats in the equatorial belt provide the opportunity to study anuran acoustic communities that remained undisturbed by human activities and possess resource levels with little long-term variation. Soundscapes without the interference of anthrophonic noises allow us to focus on the potential biophonic and geophonic constraints of the acoustic space. If stochastic competitive assembly has caused local anuran diversity, we can expect inhibitory effects of established species on recruitment to emerge by completely occupying the available niche space. Then, the niche breadth of established species is similar and the best competitors for the most frequent conditions are very abundant because they are able to prevent establishment by species similar to themselves. We chose four pristine wetlands in the Volcanoes National Park (VNP, Rwanda) to address these issues in equatorial highland anuran communities [27]. Volcanoes National Park has had strict preservation policies in place since 1929, largely because of the presence of the endangered mountain gorilla (*Gorilla beringei beringei*), and human access is currently restricted to daylight hours [28,29,30]. The inability to visit the field sites outside daylight hours is a challenge for acoustic studies of anuran communities, but we overcame this restriction using devices for Passive Automated Monitoring (PAM [31,32,33,34,35]) that allowed for 24 h daily recordings of the soundscape over seven months in four VNP wetlands (2546–3188 m altitude). 

The aims of this study were: (1) to identify the species assemblages forming local acoustic communities in wetlands differing in structure and altitude; (2) to estimate the potential limitation of acoustic space by masking biophonic and geophonic noise (wind, rain, birds, mammals, insects); (3) to measure acoustic niche breath and overlap to test the predictions of the stochastic niche theory and the ANH. 

## 2. Materials and Methods

### 2.1. Study Area

**Figure 1:**
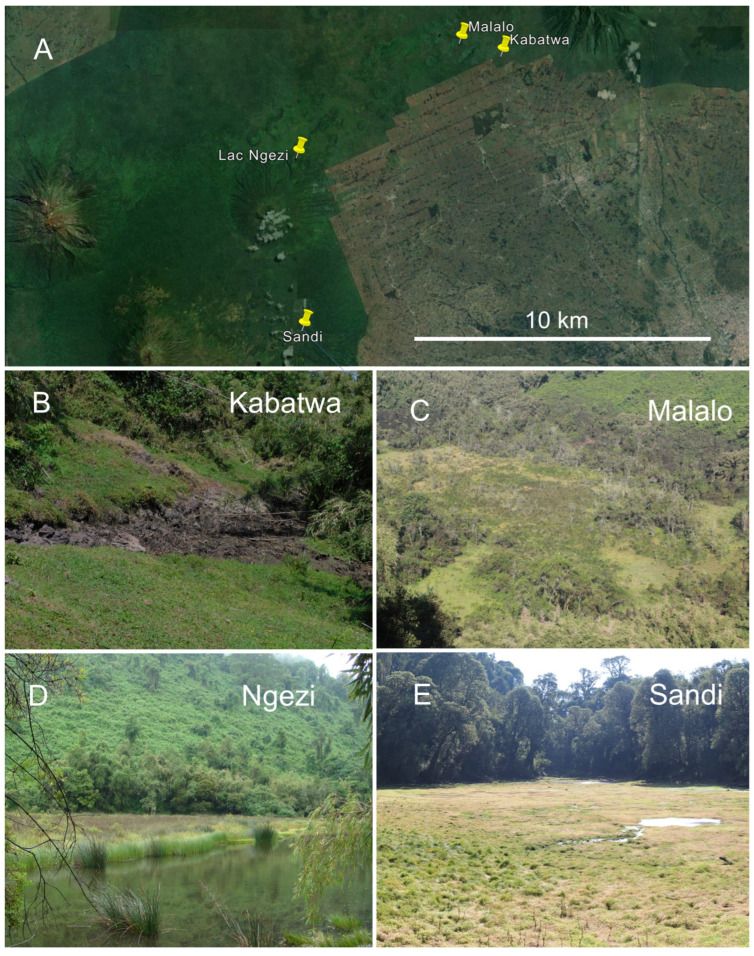
Study area. (**A**) Location of the focal wetlands within the VNP; (**B**) Kabatwa, temporary pond area, currently dry; (**C**) Malalo, swamp area within a depression (**D**) Ngezi, transition between lake and bog area; (**E**) Sandi, swamp area enlaced by forest. Source of images: (**A**) Google Earth, modified; (**B**,**C**,**E**) by D. Tuyisingize (July 2024, dry season aspect); (**D**) by U. Sinsch (March 2009, rainy season aspect).

**Figure 2:**
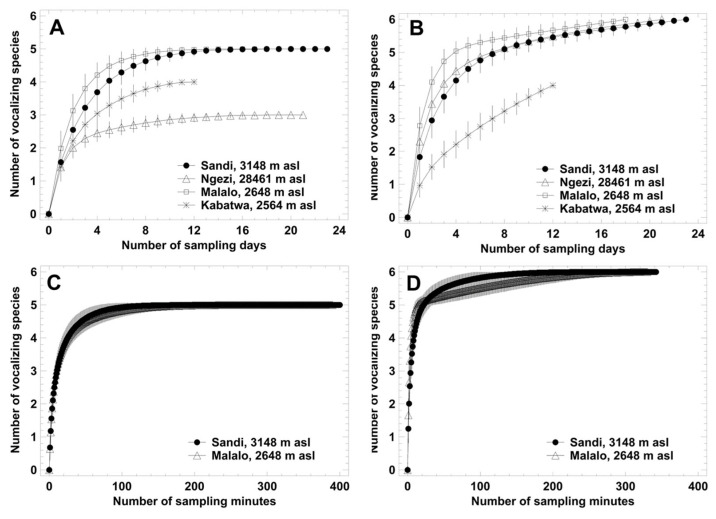
Species accumulation (rarefaction) curves based on sample days (**A**,**B**) and on 1 min samples (**C**,**D**). Left: records obtained at daylight hours (6 a.m.–6 p.m.); right: record obtained during night (7 p.m.–5 a.m.). Data are given as average estimates and the corresponding 95% confidence interval.

**Figure 3:**
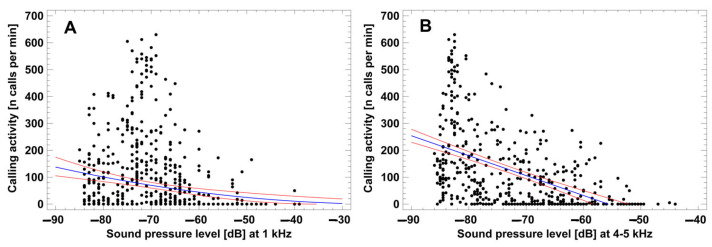
Geophonic (**A**) and biophonic noise (**B**) influence on the calling activity of *H. castaneus* in Sandi. Dots represent single sample minutes; blue and red lines represent the regression model and its corresponding 95% confidence range. For statistical details see text.

Between September 2019 and March 2020, we automatically recorded the spontaneous vocalizations given by anurans in four focal wetlands within VNP (1°21′–1°35′ S, 29°22′–29°44′ E; Figure 1A). For the acoustic diversity assessment, we chose wetlands along an altitudinal gradient of 642 m: (1) Kabatwa at 2546 m a.s.l., a small-sized wetland including a seasonal pond of about 99 m^2^ near the border of the VNP to cultivated land (1°24′29.8″ S, 29°33′41.6″ E; Figure 1B); (2) Malalo at 2648 m a.s.l., a ca. 27,000 m^2^ sized clearing in otherwise forested habitat with a permanent swamp (1°24′15.35″ S, 29°32’51.67″ E; Figure 1C); (3) Ngezi at 2861 m a.s.l., a depression of ca. 28,850 m^2^ including a permanent lake and a bog area surrounded by montane forest (1°26′32.90″ S, 29°29′38.27″ E; Figure 1D); and (4) Sandi at 3188 m a.s.l., a clearing within an otherwise forested habitat, size about 5725 m^2^ including a seasonal pond (1°29′56.8″ S 29°29′47.3″ E; Figure 1E). The line-of-sight distance between two neighboring localities varied between 1.7 km (Kabatwa–Malalo), 6.7 km (Ngezi–Sandi), and 7.2 km (Malalo–Ngezi). The wetlands represented a moisture gradient (decreasing breeding site availability), ranging from a permanent bog with large lake (Ngezi) over large swamps with several seasonal ponds (Sandi, Malalo) to a small swamp with one small seasonal pond (Kabatwa). The vegetation in VNP includes mixed forest, bamboo, *Hagenia* woodland, herbaceous, brush ridge, and subalpine and alpine zones, whereas inside the focal wetlands species such as *Juncus oxycarpus* and *Mariscus karisimbiensis* dominate, with encroachment by the dryland species *Hypericum revolutum* and *Yushania alpina* at some localities [29]. 

We recorded temperature regime at altitudes corresponding to Sandi and Malo at nearby sites (Bisoke 3119 m a.s.l., Kansoro 2474 m a.s.l.) using Tinytag Datalogger (Gemini Data Loggers Ltd, Scientific House, Terminus Road, Chichester, West Sussex, PO19 8UJ, United Kingdom), recording the air temperature at 2 m above ground in 1 h intervals.

### 2.2. Field Sampling and Study Period

At a randomly chosen location at the edge of each wetland, we installed an Automatic Waterproof Recording System Songmeter SM-4 (Wildlife Acoustics Inc., Maynard, MA, USA) ca. one meter above ground level fixed to a tree. The Songmeter has flexible programming capabilities, records in two-channel, 16-bit PCM .wav files at a sampling rate of 44.1 kHz using two built-in omnidirectional microphones, and stores files on removable 128 GB SD cards in .wav format. We set the Songmeter devices to record for the first five minutes of every hour in the focal wetlands. The recordings were automatically digitized. The SD cards and batteries were collected and replaced once a month. 

We recorded in each wetland over the course of a rainy season (September to November 2019), the dry season (December 2019 to February 2020), and the beginning of the second rainy period (March 2020) [29]. As there were only two Songmeter devices available, after recording for periods of 7–10 days, we moved them from one wetland to the next to obtain a record sample of minimum 10 days per locality and month. For analysis, we selected 24 h records obtained from 1–5 consecutive days: 17–21 December, 23–27 February, and 6 and 9 March at Kabatwa (n_total_ = 12 d); 26–27 September, 1–2 October, and 22 November, 15–18 December, 8–11 January, and 12–14, 18–19 February at Malalo (n_total_ = 18 d); 27–30 September, 4–9 October, 17–21 December, and 23–28 February at Ngezi (n_total_ = 21 d); 22 and 26–27 September, 7–8 October, 22 and 29–30 November, 17–22 December, 31 January, 1 and 23–26 February, and 19–20 March at Sandi (n_total_ = 23 d).

### 2.3. Analyses of Acoustic Records

Obtaining biological data from sound recordings is a complex task when the goal is to assess species diversity and to quantify call activity because processing large datasets is time-consuming [36]. We prepared and analyzed records manually, using Adobe Audition 1.0 to convert stereo to mono .wav files with a sample rate of 44.1 kHz at a resolution of 16 bits. We then obtained frequency information through fast Fourier transformation (FFT, width 1024 points) and created audio spectrograms using the Blackman–Harris window function (1024 bands resolution) before filtering out ambient noise outside the frequency range of anuran vocalizations below 400 Hz and above 6000 Hz. Then, we screened all records for the acoustic presence of anuran vocalizations (= percentage of records including the advertisement calls of one or more species). We distinguished between samples recorded during daylight hours, including dawn and dusk (6 a.m.–6 p.m.) and at night (7 p.m.–5 a.m.).

To associate anuran vocalizations to species, we used the spectrogram view to look for signals that (1) matched in temporal and frequency structure with species listed in the auditory library of the amphibians of Rwanda [27], (2) were clearly distinguishable from the background noise (minimum intensity threshold 5 dB, thus exceeding background noise level), and (3) were not masked in part by mammal, bird, or insect vocalizations. As *Phrynobatrachus graueri* and *Amietia ruwenzorica* occur in Virunga Massif [27], and the features of their advertisement call have not yet been described, we also visually and aurally explored the records for anuran-like signals not matching those of any of the described species. For each species detected, we calculated the specific acoustic presence (=percentage of hourly records including advertisement calls at a given wetland, distinguishing between daylight and night records). We obtained an extensive dataset to evaluate the processes shaping the local acoustic communities. The local species lists obtained by identifying the anurans vocalizing using the PAM data were cross-checked with previous visual encounter accounts at the same localities compiled between 2009 and 2017 [27,29,37]. 

The hourly 5 min records were subdivided into five 1 min sections and analyzed separately. The analysis of a single 1 min sample lasted between 2 and 17 min with an average of about 5 min. In agreement with the recommendations of de Araujo et al. [31], we opted to limit our visual and aural inspections to the first two minutes of each recording. 

For each 1 min section, we determined three parameters describing acoustic features of the local anuran community: (1) number of distinct species [n; range: 1–8]; (2) specific call rate [n/min; range 0–650] as the number of recognizable advertisement calls in each species separately (for *P. bequaerti*, we counted the number of the long notes, as the number of clicks added to the long note varied); and (3) dominant frequency [Hz; range: 1000–5000] of the first recognizable advertisement call of each species (for *P. bequaerti*, we measured the dominant frequencies of long notes and of the first click following the long note). To characterize quantitively geophonic and biophonic noise, we measured the average sound pressure level [dB] at 1 kHz (SPL_1kHz_), at 2 kHz (SPL_2kHz_), at 3 kHz (SPL_3kHz_), at 4 kHz (SPL_4kHz_), and at 5 kHz (SPL_5kHz_). The rationale for choosing frequencies from the 1–5 kHz range was that the sound pressure level of anuran advertisement calls is greatest in this frequency range. Screening the frequency-specific SPL in relation to environmental noise, we found that SPL_1kHz_ served best as a proxy of geophonic noise level at any time of the day and (SPL_4kHz_ + SPL_5kHz_)/2 as a proxy of bird calling intensity during the daylight hours. We qualitatively estimated the intensity of precipitation using a categorical index: 0 = no rain; 1 = slight but audible rainfall; 2 = heavy rain, including thunderstorms.

### 2.4. Specific Statistics of Acoustic Niche Breadth and Overlap Calculations

We estimated acoustic niche breadth and overlap among the local species at Sandi and Malalo (similar hydrological and vegetation structure) to evaluate the influence of elevation (proxy for environmental temperature and rainfall regime [38]) on the niche segregation. We considered three niche dimensions which may contribute to acoustic resource partitioning: (1) the spectral and temporal structure of advertisement call, (2) the diel variation of calling rate, and (3) the seasonal variation of calling rate. The spectral features of the advertisement call were categorized into nine resource classes, each including the number of individuals recorded within a dominant frequency interval (class width: 500 Hz; range: 500–5000 Hz). The call composition (number of notes included) provided five resource classes (width: 1 note; range: 1–5). The number of pulses per advertisement call were categorized into 11 resource classes (class widths: 3 pulses except for class 0 for the tonal calls of *H. glandicolor*, and class 11 for calls with more than 27 pulses). The total number of resource classes referring to call structure was 25. The diel niche dimension comprises 24 resource classes, representing the hours of the day, and the seasonal dimension includes seven, one class for each month within the study period. Note that the seasonal dimension at Malalo included only six resource classes because of the lack of data in March. We run ANOVAs (fixed factors: hour or month) on the raw data of each species to obtain the average call rate calls for each diel and seasonal resource class. 

We estimated the niche breadth, NB, as the inverse of Simpson’s Diversity Index [39] and standardized it to a range between 0 and 1 as NB* = (NB − 1)/(r − 1), with r = the number of resource classes. Specialists with NB* = 0 exploit only one resource class, and generalists with NB* = 1 use all resource classes. NB* was calculated for each niche dimension separately, and for the complete acoustic niche. The average niche breadth was calculated as the arithmetic mean of all single niche breadths obtained and corresponding standard deviation. Pairwise acoustic niche overlap, performed separately for the acoustic communities of Sandi and Malalo, was estimated following Colwell and Futuyama [40]. To test for resource partitioning, we compared the average measured niche overlap NO_empiric_ (n = 15) with the theoretical niche overlap NO_random_ which would occur in the absence of resource partitioning. NO_random_ was calculated as the average of 10 random number generations within the range 0 to 1. Data scattering was compared using the corresponding standard deviations of average niche overlap. To assess the influence of altitude on the acoustic resource partitioning of the species that inhabit the two localities, we ran a cluster analysis on the matrix of niche overlap using the complete linkage procedure.

### 2.5. General Statistical Analyses

Descriptive statistics depended on the outcome of the Shapiro–Wilk test for normality. Normally distributed data were described by the arithmetic mean and 95% confidence interval. If data were not normally distributed, we used median and range. To compare groups of normally distributed data, we used one-factor ANOVA (fixed factor: SPL_1kHz_ or (SPL_4kHz_ + SPL_5kHz_)/2 and three-factor ANOVA (fixed factors: locality, hour, season) and a post hoc test with Bonferroni correction. While the number of species per minute were normally distributed, the call rate data required a log10-transformation to fit normality. Note that we used log10(call rate + 1) to include records without call detection. We used regression models (linear/square root y) to describe the correlation between call intensity and the geophonic and biophonic SPL. To compare non-parametric data, we used the Mann–Whitney U-test and the Kruskal–Wallis test. The significance level was set at alpha = 0.05. All calculations were based on the procedures of the program package STATGRAPHICS centurion for Windows, version XVIII. 

We ran a Cole rarefaction analysis to calculate species accumulation curves using the statistical package EstimateS 8.20 to check whether the acoustic species detection was exhaustive [41]. Species were coded as incidence data (presence = 1; absence = 0), independent of the actual number of calls counted. We constructed presence/absence data matrices for records during daylight and night separately, using either days (all wetlands) or 1 min records (Sandi and Malalo) as sampling units. The rarefaction analyses included 100 randomizations of sample order. 

## 3. Results

### 3.1. Local Acoustic Communities: Species Assemblage

The local soundscape records included many vocalizations of anuran amphibians as well as biophonic (mammals, birds, insects) and geophonic background noise (wind, rain) (Supplementary Figure S1), whereas anthrophonic noise (human voices, airplane) was almost absent (<0.1% of records). The anuran acoustic presence was slightly but significantly biased towards the night (medians of 100% vs. 92.9%, n = 74 d; Mann–Whitney U-test, U = 4195.0, *p* < 0.001). The medians of the nightly acoustic presence did not differ significantly among the four wetlands (Kruskal–Wallis test: KT = 7.8; *p* = 0.051), but those of daylight acoustic presence did: Malalo (67.9%) = Sandi (78.6%) < Kabatwa and Ngezi (100%) (Kruskal–Wallis test: KT = 28.8, *p* < 0.001).

We identified eight distinct advertisement call types of anurans (Table 1). All were assignable to described species using the key provided by Dehling and Sinsch [27]: *Amietia desaegeri*, *Arthroleptis schubotzi*, *Hyperolius castaneus*, *H. discodactylus*, *H. glandicolor*, *Leptopelis karissimbensis*, *L. kivuensis*, and *Phrynobatrachus bequaerti*. Apart from the advertisement calls, we identified three more types of calls. Frequently, *H. castaneus* and *H. glandicolor* gave aggression calls (Supplementary Figure S1A,C). Aggressive interactions were always associated with the chorusing of conspecifics simultaneously advertising at neighboring calling sites. The third call type resembled the advertisement call of *Leptopelis karissimbensis* in most structural features and in frequency range and was made exclusively in association with the advertisement call. We did not consider these three call types for species detection or for the assessment of the specific call activity.

The specific acoustic presence varied among the wetlands, but the dominant species was *H. castaneus* at 10–98% during daylight and 88–99% during night (Table 1). Besides *H. castaneus*, *H. glandicolor* and *L. kivuensis* were ubiquitous. *Hyperolius discodactylus* were present exclusively at Ngezi where they called often at the same time as *H. castaneus* and *H. glandicolor*. *Arthroleptis schubotzi* and *Amietia desaegeri* called rarely and exclusively during night. Records were limited to one or two hours per wetland during the whole study period.

### 3.2. Local Acoustic Communities: Species Accumulation Curves

Species accumulation curves based on samples recorded during daylight reached a saturation plateau within 10–12 sampling days and within 80–120 sampling minutes, respectively (Figure 2A,C). Except for Kabatwa, the number of species advertising during night exceeded that during the day by one or two, i.e., *H. discodactylus* and the rarely detected species *Amietia desaegeri* and *Arthroleptis schubotzi* called exclusively during darkness. Species accumulation curves based on samples recorded during night did not reach a saturation plateau at any of the four wetlands, when using recording days as sample units (Figure 2B). In contrast, using minutes as a sample unit, a plateau was reached after 120 min at Malalo and 230 min at Sandi (Figure 2D). Independent of the sample unit, the rarefaction curves for Malalo reached plateaus with fewer samples than those for Sandi. 

### 3.3. Local Acoustic Communities: Geophonic and Biophonic Noises

Rainfall and associated wind (geophonic noise) caused a considerable increase in sound pressure level at all surveyed frequencies but was most pronounced at 1 kHz (Supplementary Figure S2). Thunderstorms reached SPL_1kHz_ ranging from −50 dB to −28 dB, strong to light rain from −64 dB to −50 dB, and wind without rainfall from −88 dB to −57 dB. The lowest levels of SPL_1kHz_ were around −85 dB to −88 dB, representing silence at the recording locality. Without the confounding effects of biophonic noise caused by birds, the number of vocalizing species decreased at night with increasing SPL_1kHz_ (ANOVA, F_6,495_ = 27.0, *p* < 0.001). When no species was recorded, average SPL_1kHz_ was significantly louder than in the samples in which one species called (post hoc comparison with Bonferroni correction, *p* < 0.05). In turn, when two or more species vocalized, average SPL_1kHz_ was lower than in the zero- and one-species groups (post hoc comparison with Bonferroni correction, *p* < 0.05).

Biophonic, non-anuran noise during daytime consisted of bird vocalizations (rarely those of mammals), if there was no rainfall (Supplementary Figure S3). Birds caused background noise mainly in the frequency range of 1.5–5 kHz. Using (SPL_4kHz_ + SPL_5kHz)_/2 as a proxy of bird calling intensity, we found that the number of calling anuran species was four at most, but usually none or one. The average sound pressure level was −68 dB (ANOVA, F_4,442_ = 1.4, *p* = 0.241). 

Geophonic and biophonic noise reduced or stopped the calling activity (CA) of single species, as exemplified in *H. castaneus* at Sandi (Figure 3). CA was significantly related to the SPL_1kHz_, i.e., intensity of wind and rainfall: CA = (−3.47 − 0.17 × SPL_1kHz_)^2^ (square root y regression model; R^2^ = 5.0%, n = 460, *p* < 0.001). Strong rainfall or thunderstorms significantly reduced CA during night in *H. castaneus* (141.0 vs. 90.5 calls per minute; ANOVA, F_1,491_ = 4.08, *p* = 0.044) and *L. kivuensis* (23.7 vs. 2.5 calls per minute; ANOVA, F_1,469_ = 5.75, *p* = 0.017), and stopped calling completely in *H. glandicolor*, *L. karissimbensis*, and *P. bequaerti* (1.5–5.7 vs. 0 calls per minute). 

CA was also significantly related to SPL_4+5kHz/2_, i.e., the intensity of bird vocalizations: CA = −411.9 − 7.4 × SPL_4+5kHz/2_ (linear regression model; R^2^ = 25.5%, n = 460, *p* < 0.001). Strong bird calling activity significantly increased CA during the dry daylight hours in *H. glandicolor* (23.3 vs. 41.6 calls per minute; ANOVA, F_1,335_ = 4.87, *p* = 0.028), reduced CA in *H. castaneus* (26.7 vs. 7.3 calls per minute; ANOVA, F_1,335_ = 9.74, *p* = 0.002) and *L. kivuensis* (23.7 vs. 2.5 calls per minute; ANOVA, F_1,469_ = 5.75, *p* = 0.017), and did not have a notable effect on the CA of *L. karissimbensis*, *L. kivuensis*, and *P. bequaerti* (1.5–5.7 vs. 0 calls per minute).

### 3.4. Local Acoustic Communities: Altitudinal, Seasonal and Diel Variation

**Figure 4:**
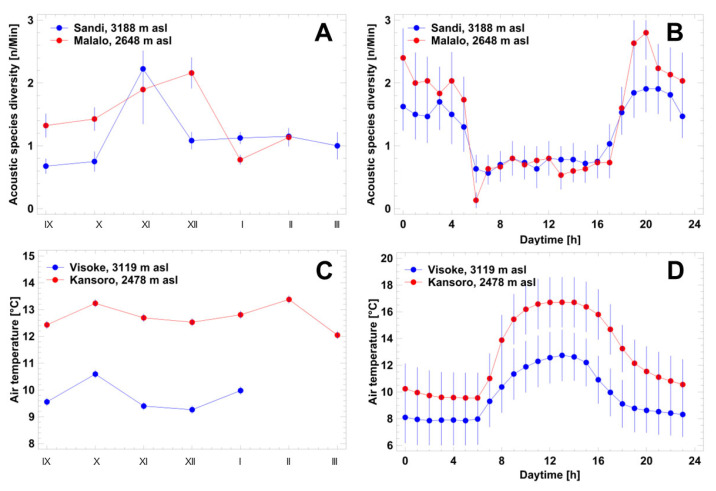
Seasonal (**A**,**C**) and diel variation (**B**,**D**) of acoustic species diversity at Sandi and Malalo. Data are given as arithmetic means with the corresponding 95% confidence interval.

Partitioning variance in acoustic species diversity by season (proxy: month), daytime (proxy: hour), and locality (Sandi, Malalo) yielded significant variation in each of the factors (Figure 4; three-factor ANOVA, *p* < 0.001). The seasonal variation of the average number of species was significant (three-factor ANOVA, F_6,1463_ = 52.7, *p* < 0.001) and reached maximum values in November (Sandi) and December (Malalo) (Figure 4A; post hoc comparison, *p* < 0.05). The number of species calling varied between daylight and nocturnal hours (Figure 4B; three-factor ANOVA, F_23,1463_ = 32.8, *p* < 0.001). Significantly more species vocalized during night (post hoc comparison, *p* < 0.05). The only exception from the rule was the average number of species calling at sunset (6 p.m.) that did not differ significantly from those during night. The calling activity in the two acoustic communities followed the same seasonal and diel pattern, but the nocturnal number of calling species was significantly greater at Malalo (three-factor ANOVA, F_1,1463_ = 64.8, *p* < 0.001). This disparity corresponded to the significantly higher seasonal and diel air temperature at the lower altitude (Figure 4C,D).

Focusing on the seasonal variation of community composition of calling anurans, we found clear differences between Malalo and Sandi (Supplementary Table S1). At Malalo, *H. castaneus* and *P. bequaerti* vocalized throughout the whole study period, whereas only *H. castaneus* was heard each month at Sandi. The specific acoustic presence of *H. castaneus* tended to increase from September to February/March in both wetlands but was usually greater in Sandi. The seasonality of the other species was less pronounced at Malalo and *H. glandicolor*, *L. karissimbensis,* and *L. kivuensis* were absent only in January.

### 3.5. Acoustic Niche Dimensions: Advertisement Call Structure

**Figure 5:**
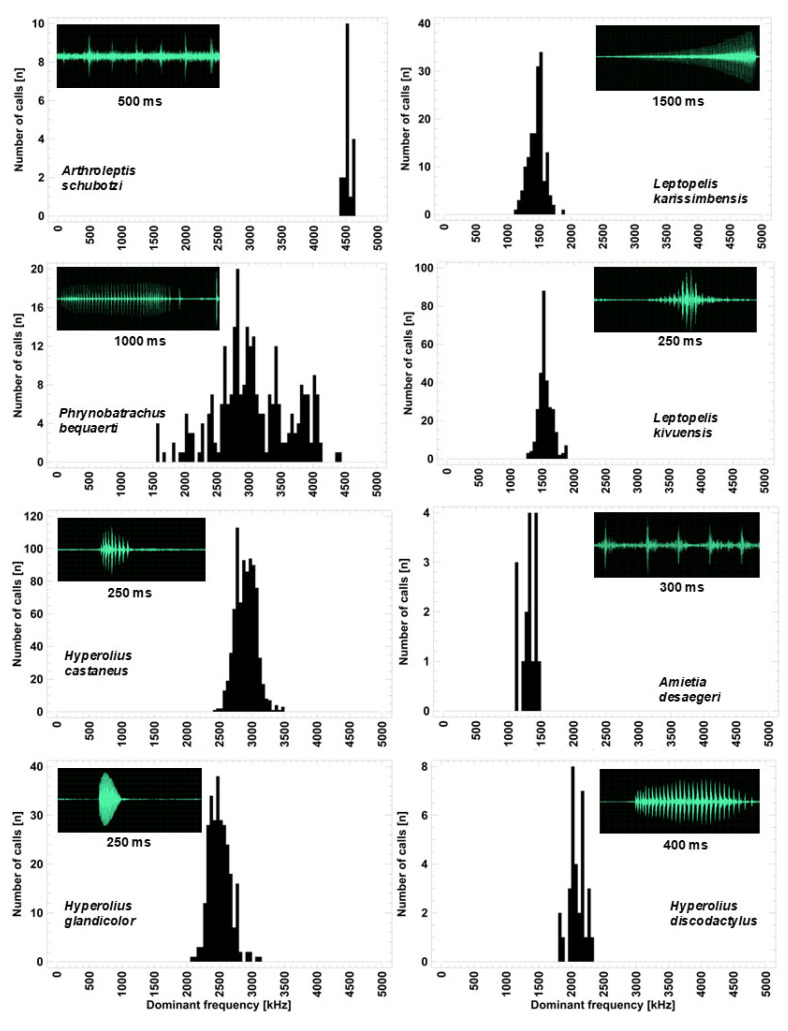
Dominant frequencies and oscillograms of advertisement calls of anuran species inhabiting the studied wetlands. (**Left side**) frequency band 2.5–4.5 kHz used by *A. schubotzi*, *P. bequaerti*, *H. castaneus*, and *H. glandicolor*; (**right side**) frequency band 1–2 kHz used by *L. karissimbensis*, *L. kivuensis*, and *A. desaegeri*; (**right side bottom**) *H. discodactylus*, exclusively present at Ngezi.

The advertisement calls of the eight species covered a frequency range from 1 kHz to 4.6 kHz (Figure 5). High-pitched calls were given by *A. schubotzi*, *P. bequaerti*, *H. castaneus*, *H. discodactylus*, and *H. glandicolor* and overlapped partially in the specific frequency range. The advertisement call of *P. bequaerti* covered a broad frequency range including two major frequency bands. The sound pressure level varied considerably between these frequency bands causing a bimodal distribution of dominant frequencies. The upper frequency band was slightly below the range of *A. schubotzi*, whereas the lower band covered completely the range of the three *Hyperolius* species. Acoustic species distinction was warranted by the temporal structure of calls with pulsed calls in *P. bequaerti* (call duration 240–980 ms, pulses of a long note numbered 4–29, followed by 1–5 short clicks), in *H. castaneus* (call duration 26–73 ms, number of pulses 6–11) and in *H. discodactylus* (call duration 169–408 ms, number of pulses 19–51), whereas *H. glandicolor* produced a tonal click (call duration 39–77 ms). This holds also true for Ngezi, the only locality in which three *Hyperolius* species occurred and called syntopically.

Low-pitched calls were given by *L. karissimbensis*, *L. kivuensis*, and *A. desaegeri*, and overlapped considerably in frequency range. Again, acoustic species distinction was warranted by the temporal structure of calls with very long pulsed calls and rising amplitude for *L. karissimbensis* (call duration 534–1444 ms, number of pulses 56–156 pulses), and short calls for *L. kivuensis* (call duration 45–83 ms, 5–12 pulses) and for *A. desaegeri* (duration 253 ms, 5 evenly spaced pulses). 

### 3.6. Acoustic Niche Dimensions: Seasonal and Diel Variation of Calling Activity

**Figure 6:**
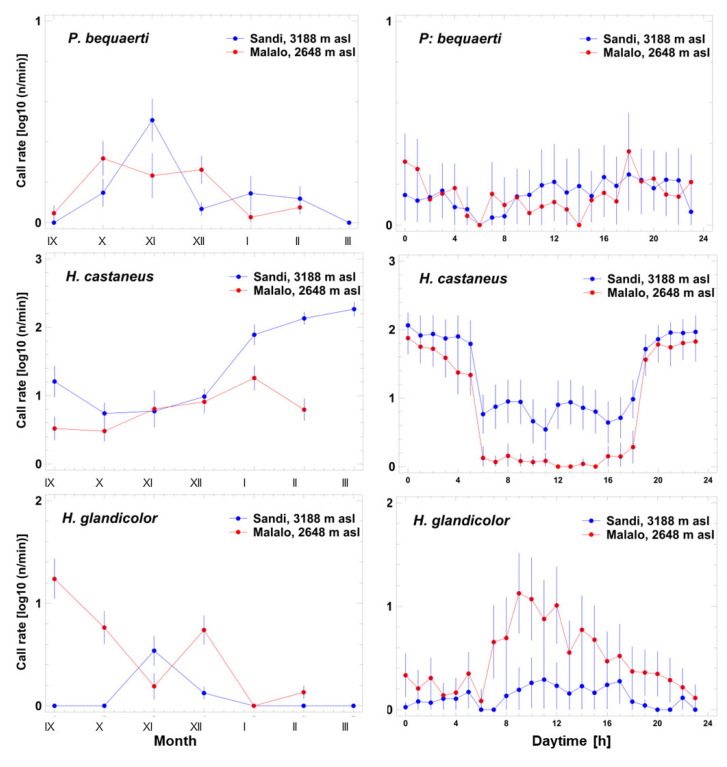
Seasonal (**left side**) and diel variation (**right side**) of log10-normalized call rate of three species with high-pitched advertisement calls at the Sandi and Malalo. Data are given as arithmetic means and the corresponding 95% confidence interval.

For the species with high-pitched advertisement calls, data on *A. schubotzi* originated from only two individuals giving a total of 17 calls and were recorded within two minutes during a night with very little background noise at Malalo. The sound pressure level of these calls was still only 14 dB at most above the background noise at the dominant frequency of 4.5 kHz. We assume that the recorded individuals called from the leaf litter of the nearby forest (natural habitat [27]), not from the wetland area near the recorder location. As *Hyperolius discodactylus* was not recorded in either Malalo or Sandi, we did not obtain data for the analyses of seasonal and diel variation of calling activity. Consequently, the quantitative analyses focused on *P. bequaerti*, *H. castaneus*, and *H. glandicolor* (Figure 6).

*Phrynobatrachus bequaerti* vocalized at any time of day and night with about the same intensity (three-factor ANOVA; F_23,1416_ = 2.2, *p* = 0.001), except for dawn (6 a.m., fewer calls than average, *p* < 0.05) and dusk (6 p.m., more calls than average, *p* < 0.05). Call rate did not differ between the two localities (three-factor ANOVA; F_1,1416_ = 2.7, *p* = 0.098). The seasonal pattern included minimum calling activity at the beginning and the end of the study period and maximum activity in November and December (three-factor ANOVA; F_6,1416_ = 32.8, *p* < 0.001). 

Among all species detected, *H. castaneus* was the one with the highest calling rate. Although these frogs were present at night and day during the whole study period in the two localities, calling activity was highly seasonal in terms of quantity (three-factor ANOVA; F_6,1352_ = 108.9, *p* < 0.001). Calling activity was low between September and December and increased to maximum values at the end of the study period between January and March. The diel calling rate showed a two-tier pattern with minimum values during daytime including dawn and dusk and maximum nocturnal values (three-factor ANOVA; F_23,1352_ = 77.7, *p* < 0.001). At Sandi, the calling rate significantly exceeded that at Malalo (three-factor ANOVA; F_1,1352_ = 209.9, *p* < 0.001). Moreover, frequent calling during the daylight hours was restricted to Sandi. 

The local, seasonal, and diel calling pattern of *H. glandicolor* was reverse to that of *H. castaneus*. The calling rate was higher in Malalo, whereas in Sandi frogs were present, but with very low calling activity (three-factor ANOVA; F_1,1408_ = 183.5, *p* < 0.0001). At the low-altitude site, most of the calling activity of *H. glandicolor* was recorded during the first half of daytime, i.e., frogs were diurnal, whereas at Sandi the low activity was fairly evenly distributed over the whole day (three-factor ANOVA; F_23,1408_ = 6.7, *p* < 0.001). The seasonal activity pattern showed maximum activity in September decreasing to lower levels from December to March (three-factor ANOVA; F_6,1408_ = 5.9, *p* < 0.001). 

**Figure 7:**
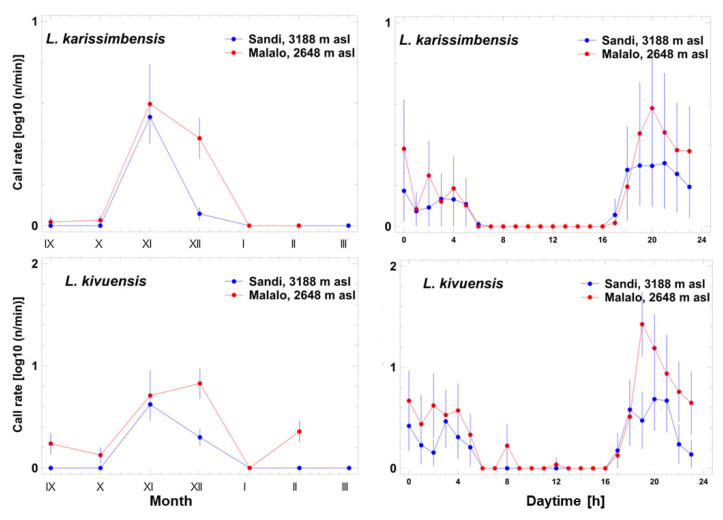
Seasonal (**left side**) and diel variation (**right side**) of log10-normalized call rate of two species with low-pitched advertisement calls at the Sandi and Malalo. Data are given as arithmetic means and the corresponding 95% confidence interval.

Among the three species pertaining to the group with low-pitched advertisement calls, data on *A. desaegeri* originated from one individual recorded at Ngezi and two at Sandi, providing a total of 15 calls in early October. Due to the scarcity of data, the analyses of seasonal and diel calling patterns were limited to the two *Leptopelis* species. The calling pattern of *L. karissimbensis* and *L. kivuensis* resembled each other with respect to season and diel variation but differed in the calling rate that was considerably greater in *L. kivuensis* (Figure 7). Most seasonal calling activity was recorded from September to December, reaching a maximum in November and December (*L. karissimbensis*: three-factor ANOVA; F_6,1380_ = 91.7, *p* < 0.001; *L. kivuensis*: three-factor ANOVA; F_6,1326_ = 67.4, *p* < 0.001). Diel calling activity started short before dusk and lasted the whole night long (*L. karissimbensis*: three-factor ANOVA; F_23,1380_ = 14.1, *p* < 0.001; *L. kivuensis*: three-factor ANOVA; F_23,1326_ = 25.2, *p* < 0.001). Nocturnal calling activity was more intense at Malalo in both species (*L. karissimbensis*: three-factor ANOVA; F_1,1380_ = 50.2, *p* < 0.001; *L. kivuensis*: three-factor ANOVA; F_1,1326_ = 111.6, *p* < 0.001).

### 3.7. Acoustic Niche Breadth and Overlap

Standardized niche breadth NB*_call structure_ showed high to moderate specialization among all species ranging from 0.07 to 0.21 with an average of 0.15 ± 0.10 at Sandi, and from 0.08 to 0.35 with an average of 0.13 ± 0.04 at Malalo (Table 2). Except for the niche of *P. bequaerti*, specific niche breadth did not differ between the two localities. Assuming an equally partitioned niche space among the six species at each locality, average niche breadth would amount to 0.17. The 95% confidence interval of the average empiric niche breadth included the expected niche breath without partitioning. NB*_diel_ and NB*_seasonal_ values were far more heterogeneous than NB*_call structure_ values, including wide breadth ranges (0.43–0.83) in *H. castaneus* and very narrow ranges (0–0.04) in *A. desaegeri* and *A. schubotzi* (Table 2). Combining the three dimensions of the acoustic niche to calculate NB*_Total_ resulted in narrow niche breadth (Sandi: 0.06–0.38, Malalo: 0.05–0.31), mainly due to the contribution of the species-specific advertisement call structure. Average NB*_Total_ was about 0.21 at the two localities.

Pairwise standardized niche overlap among the species in Sandi did not differ significantly from a normal distribution (Shapiro–Wilk test, W = 0.93, *p* = 0.319). Neither did that obtained in Malalo (Shapiro–Wilk test, W = 0.96, *p* = 0.655). The average niche overlap was NO_empiric_ = 0.35 ± 0.16 in Sandi and NO_empiric_ = 0.36 ± 0.15 in Malalo, respectively (Table 3). In both localities, the niche overlap was largest between *L. karissimbensis* and *L. kivuensis* (0.69/0.74), and smallest between *P. bequaerti* and *A. desaegeri* (0.07) in Sandi and *A. schubotzi* in Malalo (0.14). The calculation of random niche overlap NO_random_ (i.e., absence of resource partitioning) yielded values varying between 0.44 ± 0.07 and 0.53 ± 0.11 with a second-order mean of 0.49 ± 0.09. Average measured niche overlap NO_empirical_ was significantly lower than random niche overlap NO_random_ (second-order mean; Sandi: *t*-test, t = –4.76, *p* < 0.001; Malalo: *t*-test, t = –4.52, *p* < 0.001) and standard deviation of the empirical distribution as a measure of scatter was also lower (Sandi: F-test, F = 3.21, *p* = 0.002; Malalo: F-test, F = 2.93, *p* = 0.004).

**Figure 8:**
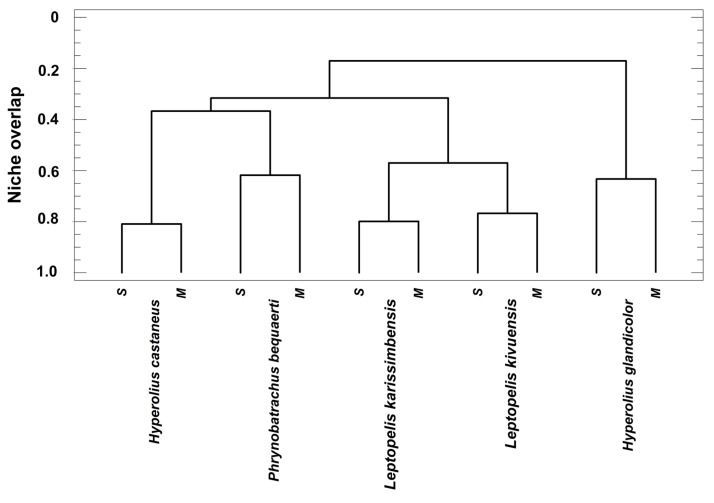
Cluster analysis (method: complete linkage) of altitudinal niche overlap of the five species occurring at Sandi (S) and Malalo (M). Standardized total breadth of the acoustic niche and of its dimensions (call feature, diel, and seasonal variation) are summarized in Table 2.

To explore the altitudinal overlap of the standardized acoustical niches, we pooled the local data originating from the five species, which occur in both Sandi and Malalo. As expected, the niches of the same species at distinct localities were more similar to each other than those of distinct species (Figure 8). The altitudinal niche overlap between conspecific niches averaged NO_empiric_* = 0.73 ± 0.09, i.e., considerably less than the expected 1.0 in the absence of niche differentiation in similarly structured habitats at distinct altitudes. In *P. bequaerti*, altitudinal niche overlap amounted to 0.62, whereas that in *H. castaneus* to 0.81.

## 4. Discussion

The equatorial highland communities inhabiting four pristine wetlands in the Volcanoes National Park (Rwanda) included four to six anuran species each indicating local species saturation, and three species were very abundant suggesting similar assembly rules, as predicted by the stochastic niche theory [12]. The acoustically detected species are eight in sum, i.e., considerably less than the 15 species reported for the whole Virunga Massif [27]. The discrepancy is because the latter account is a taxonomic list including species limited to the large Rugezi wetland (e.g., *Ptychadena chrysogaster*, *Xenopus wittei*). 

Focusing on the functional units of the ecosystem, the anuran communities inhabiting the four focal wetlands, and we have comparable information on species composition obtained in visual encounter surveys since 2009 [27,29,37]. Table 4 contrasts the results of active searches (AS) during the past decade with those of seven months of passive acoustic monitoring (PAM). AS also yields chance sightings of non-reproductive individuals that disperse or forage, while PAM identifies the species advertising and therefore the intention to reproduce and to compete for the local acoustic space [6,42,43]. For example, the few specimens of *Sclerophrys kisoloensis* occasionally observed at Sandi and Ngezi apparently do not breed there as the unconfoundable and loud advertisement calls of these toads were never heard. The misidentification of species is another issue of AS because several species groups include morphologically similar species, which often require a reliable identification comprising a more detailed inspection than possible in the field (Table 4). Species of the *Hyperolius viridiflavus* group are challenging to identify only based on external color patterns; therefore, genetic or bioacoustics determination is mostly necessary [44]. *Amietia* spp. are subject to ongoing taxonomic revisions, so that confusion of *A. nutti* with *A. desaegeri* is most probable [37,45]. Bioacoustic detection may be imperfect because we were only able to record the low-amplitude calls at short distances from the Songmeters. *Arthroleptis* spp. are leaf-litter frogs that live in montane forests and *A. adolfifriederici* has been found in the area [46]. Bioacoustic detection requires them to call from the edge of the forest near the recorder position at the wetland. The recording of *A. schubotzi* in this study was such a fortunate occasion. The apparent absence of *P. graueri* may be because its advertisement call is still undescribed, and calls may have been masked or not recognized. Alternatively, this species has been confused with the similar *P. bequaerti* in previous surveys [27]. 

### 4.1. Do Altitude and Wetland Structure Constrain the Species Assembly of Local Acoustic Communities?

Equatorial high-altitude habitats are humid and permanently provide diverse water bodies for the reproduction of anurans [47]. This holds true for the tropical Andes as well as for the Virunga Massif, but the overall species diversity of anurans inhabiting elevations of at least 3000 m a.s.l. is considerably higher in the Neotropics than in the Afrotropics ([27,29,47] and this study). Focusing on the scale of communities, and as predicted by the stochastic niche theory, the acoustic space is occupied by almost the same number of species per locality, 2–7 species in the equatorial Andes (2660–3400 m a.s.l.), and 4–6 species in the Virungas, indicating that constraints of niche space are similar ([48,49] and this study). 

Our study demonstrates that different wetlands, and associated acoustic communities, include the same dominant species (*H. castaneus*, *H. glandicolor*, *L. kivuensis*). This suggests that assembly rules and environmental filtering are similar in different altitudes and wetland structure. Spatial distance among neighboring wetland amounts to more than 6 km (except for Kabatwa–Malalo), which renders dispersal as a source of local species homogenization unlikely [50]. As predicted by the stochastic niche theory, the rarely present *S. kisoloensis* is apparently unable to establish breeding populations in the local communities. Moreover, 600 m altitudinal difference between Kabatwa and Sandi translates into an average temperature difference of 5 °C during day and 2 °C during night, indicating that these species possess a remarkable thermal tolerance range of reproductive behavior. In fact, in a similar altitudinal gradient (2900–5500 m a.s.l.), three neotropical frog species showed similar diel patterns of call activity, supporting the hypothesis that temperature plays a minor role in the colonization of highland habitats by these species [51]. 

While species numbers did not differ among similar-sized wetlands, local species composition did, because a permanent lake like Lac Ngezi provided an additional habitat type (presence of *H. discodactylus*, absence of *P. bequaerti*). In contrast, the structurally similar wetlands of Sandi and Malalo were comparable in species composition except for the alternative occurrence of a single rare species, which suggests that habitat structure is more relevant for species assembly than elevation itself. In conclusion, the acoustic space of high-altitude communities in the VNP provides niches for about six anuran species.

### 4.2. Do Geophonic and Biophonic Noise Constrain Acoustic Niche Space of Anuran Species? 

In tropical regions, the frequent heavy rainfalls are among the loudest natural sources of geophonic noise affecting the entire frequency range of anuran vocalizations. As expected, the calling activity of neotropical anurans is strongly affected by rainfall [20], which agrees with the findings in this study. The general response (reduction or stopping of calling) suggests that the local species tend to reduce energetic costs for signal production during short periods of disturbance, i.e., species opt for temporal avoidance behavior. Consequently, anurans continue to use the same acoustic niche space that is covered by the geophonic noise.

From the point of view of anurans, the vocalizations of other taxa are biophonic noise with the potential to mask a part of the frequency range and so constrain acoustic space. In the soundscape of the VNP, bird vocalizations were dominant in loudness and frequency coverage and overlapped with the complete frequency range of the local anurans. As the presence of loud bird noise is predictable in time (from sunrise to sunset, during night few birds call), we expected a strong selective pressure to avoid the daylight hours for anuran calling. Our study did not prove this expectation because while calling anurans were indeed more frequent during night than during day, three species (*H. castaneus*, *H. glandicolor*, *P. bequaerti*) produced a significant number of calls during the daylight hours, indicating that biophonic noise does not generally hinder acoustic communication in high-altitude wetlands and cannot be considered a niche constraint. Thus, the geophonic and biophonic background noises in pristine habitats may reduce the calling activity of some anuran species or silence calling completely in others, but do not affect the volume of the acoustic niche space. 

### 4.3. Does Stochastic Niche Theory Predict Realized Acoustic Niche Breath and Overlap? 

Most studies on the ecological niches of anurans have dealt with the trophic biology of sympatric species in lowland habitats, far less with highland communities competing for acoustic space [52,53,54,55]. The acoustic space of mixed-species assemblages is thought to reflect selective pressures against signal interference and degradation but also phylogenetic relationships among species [56,57,58]. In this study, we consider the niches within the acoustic space to integrate advertisement call features, diel, and seasonal variation in calling activity [4,16,59,60,61]. For example, the acoustic niches of congeners (*Eleutherodactylus* spp.) show the partitioning of the frequency coverage as predicted by the ANH, but little segregation of diel calling activity [14]. Anurans of Australian lowland communities and an African medium-elevation community also partition acoustic space by spectral features of the call [4,62]. Hence, current evidence suggests that acoustic competition among syntopic anurans is mainly reduced by minimizing the frequency overlap of advertisement calls, whereas diel or seasonal partitioning of call activity is of minor importance, probably due to the continuous presence of water bodies to breed. 

In agreement with the stochastic niche theory, niche breadth with respect to call features is very similar among all species of Rwandan high-elevation communities, leading to a complete coverage of the frequency range (1–5 kHz) in the available acoustic space. This is a reliable indicator that the local acoustic community is species-saturated [12]. In a medium-elevation community in Rwanda, which differed completely in species number and taxon composition from the high-elevation communities, niche breadth distribution also indicated stochastic competitive species assembly [4]. The temporal dimensions of the acoustic niches entered a wider scattering of niche breadth among species, suggesting that species use distinct time slots for calling as a second mechanisms to reduce acoustic competition. As species composition is fairly similar among all high-elevation communities studied and suitable advertisement/breeding habitats are available all year round, the local species seem to occupy the entire available acoustic niche space, probably preventing the establishment of additional species. The predicted low invasibility seems to be a common feature in the studied communities.

Realized overlap among acoustic niches is expected to be smaller than average in a random distribution of niche breadths, reflecting the selective pressure to reduce competition [61]. The prediction holds true for the overlap of climatic and trophic niches in montane neotropical species [48,63]. We provide evidence for this prediction in high-elevation acoustic communities (this study) and in those at medium elevations [4]. If the estimate of niche overlap focusses on the diel and seasonal dimensions of calling activity in equatorial lowland communities, overlap was instead greater than expected, possibly supporting the minor role of temporal niche dimensions for acoustic partitioning [61]. The contribution of the temporal dimensions to niche differentiation increases in communities with almost the same species set (Sandi, Malalo) along elevational gradients, because conspecific niches do not differ with respect to call features but vary considerably in diel and seasonal call activity. Evaluating all available evidence, stochastic niche theory quite accurately predicts the realized niche breaths and overlap in acoustic communities. 

## 5. Conclusions

Acoustic communities of equatorial anuran species in pristine Afromontane high-elevation habitats (2500–3500 m a.s.l.) share several features with those in the Neotropics ([48,63] and this study): (1) they provide niches for 4–7 species, depending on the wetland structure; (2) species composition is similar independent of elevation, including a common stock of three species; (3) *Hyperolius castaneus* proved to be an umbrella species for the high montane wetland communities in Rwanda, the DR Kongo, and Uganda [37]; (4) niche breadth with respect spectral and temporal call features is similar in all local species and is not influenced by difference in elevation of the wetland; and (5) niche breadth with respect to the diel and seasonal variation of call activity varies according the local temperature regime at different altitudes. 

In agreement with the acoustic niche hypothesis, vocal competition among the established species is greatly reduced by the specific features of the advertisement call, namely the use of distinct frequency ranges for communication. Yet, the ANH does not explain the limited number of species per community and the dominance of three very abundant species at all studied localities. The stochastic niche theory fills this gap by highlighting that species saturation is the result of the complete occupation of the available niche space by a limited number of species with about the same niche breadth. As they are the best competitors for the acoustic space within their niches, they are very abundant and prevent the establishment of similar species [4,12]. Consequently, niche overlap is lower than expected in random distribution. In conclusion, we demonstrate that the theoretical limitations of the ANH are resolved by the wider framework of the stochastic niche theory describing properly the features of acoustic communities of anurans.

## Figures and Tables

**Table 1:** Species diversity and specific acoustic presence [%] in four anuran communities at the VNP. Specific acoustic presence = percentage of hourly records including advertisement calls in a given wetland, distinguishing between daylight and night records. Abbreviations: D = daylight 6 a.m.–6 p.m.; N = night 7 p.m.–5 a.m.

Species		Kabatwa 2546 m a.s.l.	Malalo 2648 m a.s.l.	Ngezi 2861 m a.s.l.	Sandi 3188 m a.s.l.
*Amietia desaegeri*	D N	-	-	0% 1.0%	0% 1.5%
*Arthroleptis schubotzi*	D N	-	0% 0.6%	-	-
*Hyperolius castaneus*	D N	79.2% 88.3%	10.0% 90.9%	98.1% 99.0%	50.7% 94.4%
*Hyperolius discodactylus*	D N	-	-	0% 21.3%	-
*Hyperolius glandicolor*	D N	0% 1.1%	36.7% 22.7%	24.1% 83.5%	10.0% 6.1%
*Leptopelis karissimbensis*	D N	-	1.5% 20.8%	0% 6.8%	2.5% 17.8%
*Leptopelis kivuensis*	D N	2.8% 25.0%	5.1% 52.1%	0.9% 29.1%	3.7% 26.0%
*Phrynobatrachus bequaerti*	D N	4.1% 3.3%	18.5% 26.0%	-	13.9% 18.4%

**Table 2:** Standardized niche breadth NB* of the single dimensions (call structure, diel, and seasonal) of the acoustic niche. NB*_total_ is the breadth of the complete niche using an additive calculation model. Abbreviations: S = Sandi; M = Malalo.

Species		NB*_Call structure_	NB*_Diel_	NB*_Seasonal_	NB*_Total_
*Amietia desaegeri*	S	0.12	0.04	0.00	0.06
	M	-	-	-	
*Arthroleptis schubotzi*	S	-	-	-	-
	M	0.12	0.00	0.00	0.05
*Hyperolius castaneus*	S	0.14	0.65	0.55	0.38
	M	0.13	0.43	0.83	0.31
*Hyperolius glandicolor*	S	0.11	0.35	0.08	0.17
	M	0.11	0.43	0.26	0.29
*Leptopelis karissimbensis*	S	0.07	0.30	0.02	0.12
	M	0.08	0.27	0.19	0.14
*Leptopelis kivuensis*	S	0.14	0.31	0.11	0.24
	M	0.14	0.30	0.38	0.26
*Phrynobatrachus bequaerti*	S	0.35	0.63	0.25	0.35
	M	0.21	0.64	0.55	0.28

**Table 3:** Pairwise standardized niche overlap of six species among the species comprising the anuran communities at Sandi and Malalo. Range of niche overlap: 0 to 1.0.

	*H. castaneus*	*H. glandicolor*	*L. karissimbensis*	*L. kivuensis*	*P. bequaerti*
Species at Sandi					
*Amietia desaegeri*	0.2398	0.1211	0.2822	0.2679	0.0741
*Hyperolius castaneus*		0.2884	0.3214	0.4050	0.3856
*Hyperolius glandicolor*			0.4301	0.4596	0.4535
*Leptopelis karissimbensis*				0.7389	0.3895
*Leptopelis kivuensis*					0.3710
Species at Malalo					
*Arthroleptis schubotzi*	0.2585	0.1799	0.2388	0.3738	0.1422
*Hyperolius castaneus*		0.3339	0.4678	0.5766	0.4402
*Hyperolius glandicolor*			0.2190	0.2565	0.3455
*Leptopelis karissimbensis*				0.6898	0.4275
*Leptopelis kivuensis*					0.4185

**Table 4:** Species diversity in four anuran communities at the VNP, assessed using active search during daylight, 2009–2017 (AS [27,29,37]) or passive acoustic monitoring, 2019–2020 (PAM, this study). ^1^ misidentified as *A. nutti*; ^2^ misidentified as *H. viridiflavus* or *H. cinnamomeoventris*; ^3^ not distinguished between *L. karissimbensis* and *L. kivuensis*; ^4^ misidentified as *P. graueri* or *P. parvulus*.

Species	Kabatwa		Malalo		Ngezi		Sandi	
	AS	PAM	AS	PAM	AS	PAM	AS	PAM
*Amietia desaegeri*	-	-	-	-	X ^1^	X	-	X
*Arthroleptis schubotzi*	-	-	-	X	-	-	-	-
*Hyperolius castaneus*	X	X	X	X	X	X	X	X
*Hyperolius discodactylus*	-	-	-	-	X	X	-	-
*Hyperolius glandicolor*	X ^2^	X	X ^2^	X	X ^2^	X	X ^2^	X
*Leptopelis karissimbensis*	-	-	X ^3^	X	X ^3^	X	X ^3^	X
*Leptopelis kivuensis*	X ^3^	X	X ^3^	X	X ^3^	X	X ^3^	X
*Phrynobatrachus bequaerti*	X ^4^	X	X ^4^	X	X ^4^	-	X ^4^	X
*Sclerophrys kisoloensis*	-	-	-	-	X	-	X	-

## Data Availability

Original acoustic records are available on request by the authors (D. Tuyisingize c/o Dian Fossey Gorilla Fund, Ellen DeGeneres Campus, Kinigi, Rwanda; dtuyisingize@gorillafund.org). Original sample .wav files (Kabatwa, 17 December 2019, 5–9 p.m.; Malalo, 22 November 2019, 5–9 p.m.; Ngezi, 17 December 2019, 5–9 p.m.; Sandi, 29 November 2019, 5–9 p.m.) are publicly available at Zenodo doi: 10.5281/zenodo.13203104.

## Supplementary Materials

The following supporting information can be downloaded at: https://www.mdpi.com/article/10.3390/ani14162360/s1. Table S1: Monthly specific acoustic presence of the anuran species in two wetlands in the VNP. Figure S1: Examples of the nocturnal soundscape with anuran vocalizations at the studied localities. (A) Kabatwa; (B) Malalo; (C) Ngezi; (D) Sandi. The sonograms of advertisement call are labelled with species names and white arrows. The yellow arrow indicates the aggression call of *H. castaneus*. Figure S2: Geophonic noise (sound pressure level at 1 kHz) and anuran species calling during nighttime (7 p.m.–5 a.m.). (A) Noise range and anuran species calling within one minute. Noise levels are given as minimum, maximum, and average and the corresponding 95% confidence interval. (B) Audio spectrogram (left) and power spectrum (0.5–6 kHz, right) of background noise level at −84 dB, i.e., almost silence. (C) Audio spectrogram (left) and power spectrum (0.5–6 kHz, right) of background noise levels at −40 to −28 dB during a thunderstorm with heavy rainfall. Figure S3. Biophonic noise (sound pressure level at 1 kHz) and anuran species calling during daylight (6 a.m.–6 p.m.) at hours without rainfall. (A) Noise range and anuran species calling within one minute. Noise levels are given as minimum, maximum, and average and the corresponding 95% confidence interval. (B) Audio spectrogram (left) and power spectrum (0.5–6 kHz, right) of background noise level at −76 dB, i.e., few birds calling at a distance. (C) Audio spectrogram (left) and power spectrum (0.5–6 kHz, right) of background noise levels at −58 to −48 dB, i.e., many birds calling from short distance.
