# Supplementary material for: Species Assembly of Highland Anuran Communities in Equatorial Africa (Virunga Massif): Soundscape, Acoustic Niches, and Partitioning
Source: Animals (Basel). 2024 Aug 15;14(16):2360. doi: 10.3390/ani14162360 (PMC11350915; doi:10.3390/ani14162360)
Supplement: Supplementary file 1 [file animals-14-02360-s001.zip › animals-3132222-supplementary.pdf]

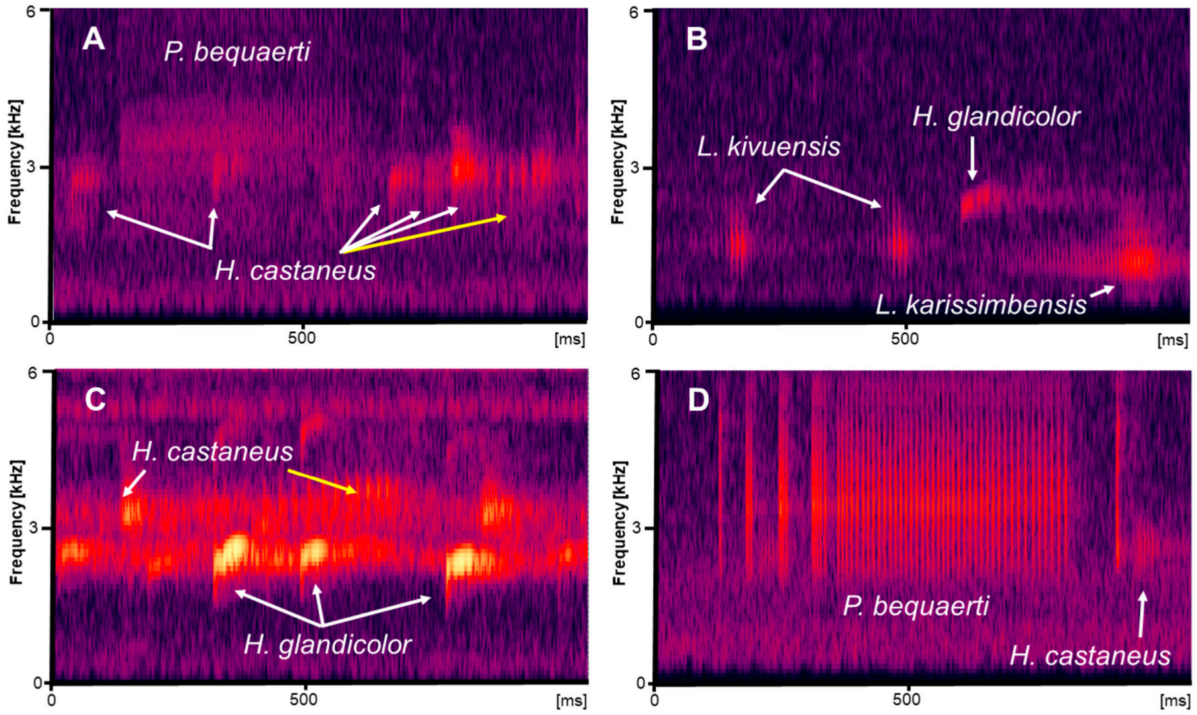

Figure S1. Examples of the nocturnal soundscape with anuran vocalizations at the studied localities. **(A)** Kabatwa; **(B)** Malalo; **(C)** Ngezi; **(D)** Sandi. The sonograms of advertisement call are labelled with species name and white arrows. The yellow arrow indicates the aggression call of *H. castaneus*.

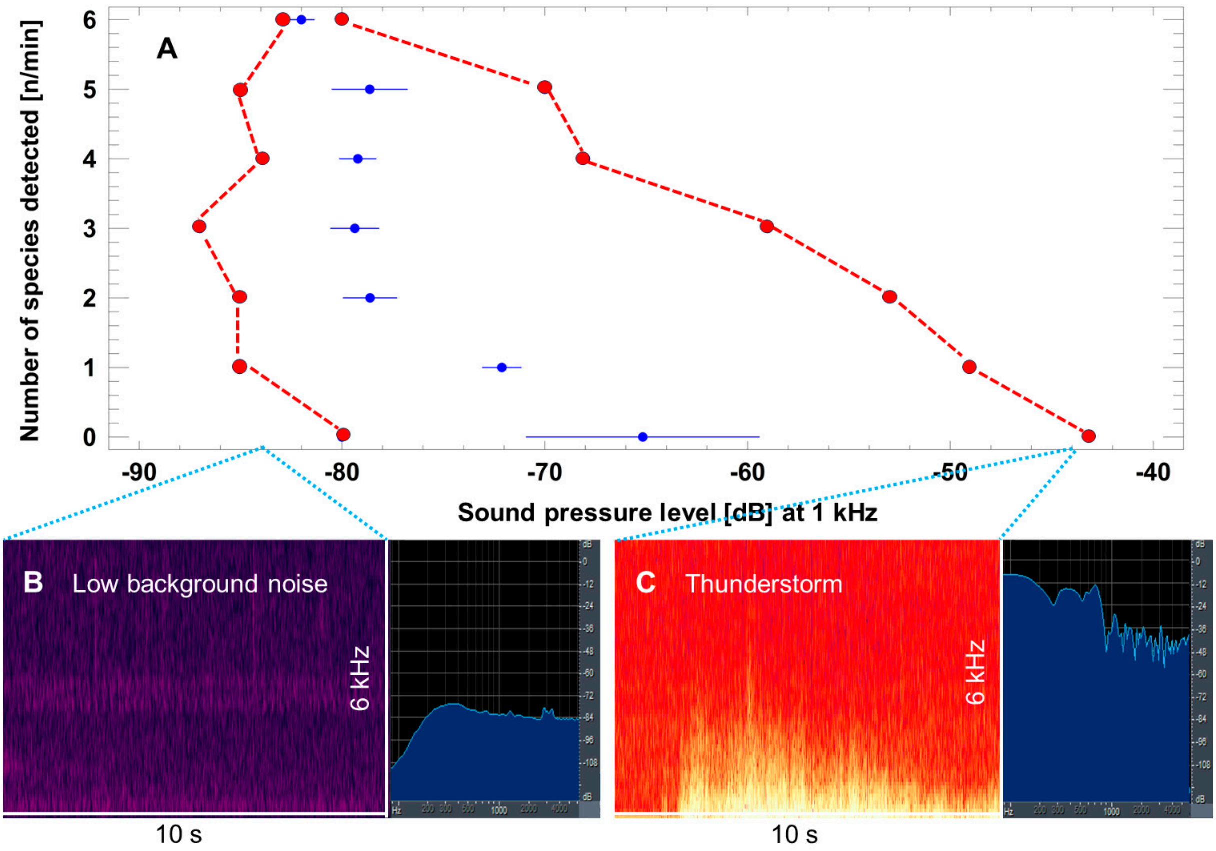

Figure S2. Geophonic noise (sound pressure level at 1 kHz) and anuran species calling during nighttime (7 pm – 5 am). **(A)** Noise range and anuran species calling within one minute. Noise levels are given as minimum, maximum and average and the corresponding 95% confidence interval. **(B)** Audio spectrogram (left) and power spectrum (0.5 – 6 kHz, right) of background noise level at -84 dB, i.e., almost silence. **(C)** Audio spectrogram (left) and power spectrum (0.5 – 6 kHz, right) of background noise levels at -40 to -28 dB during a thunderstorm with heavy rainfall.

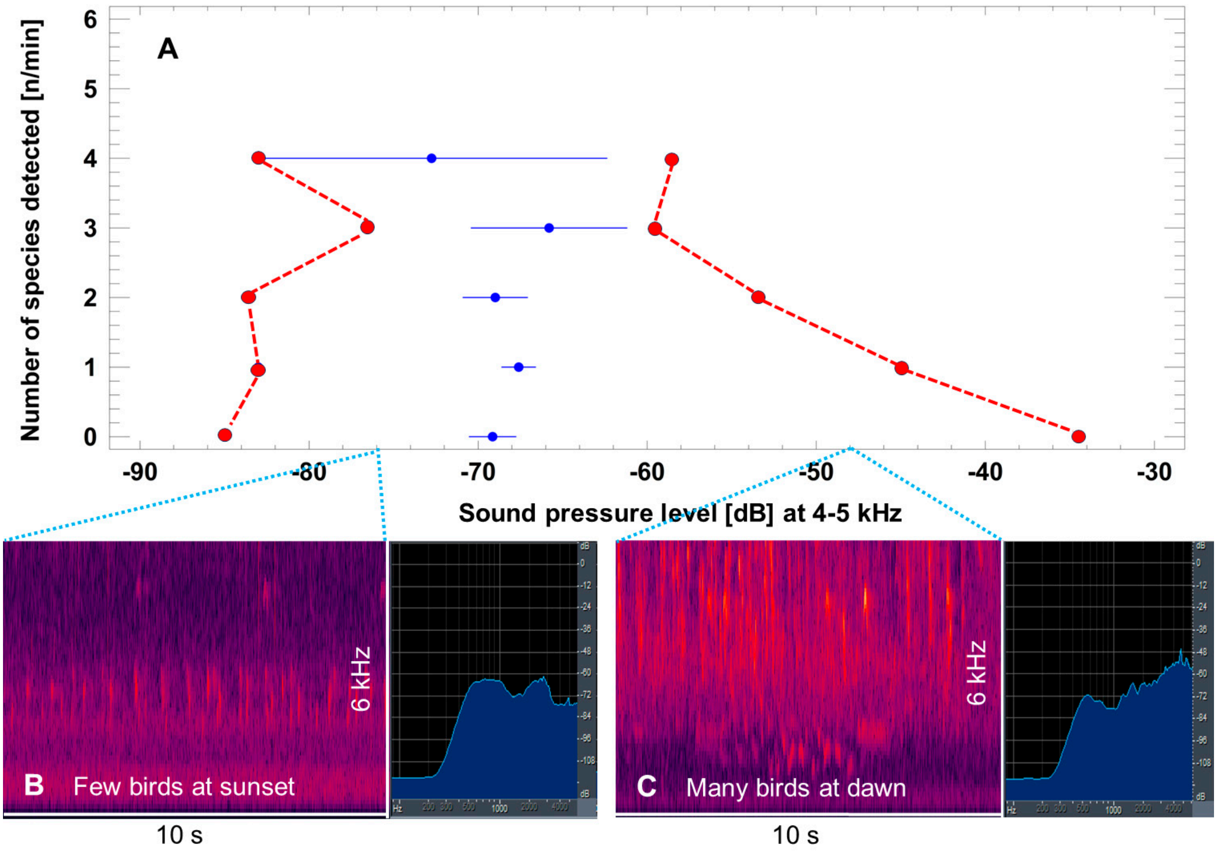

Figure S3. Biophonic noise (sound pressure level at 1 kHz) and anuran species calling during daylight (6 am – 6 pm) at hours without rainfall. **(A)** Noise range and anuran species calling within one minute. Noise levels are given as minimum, maximum and average and the corresponding 95% confidence interval. **(B)** Audio spectrogram (left) and power spectrum (0.5 – 6 kHz, right) of background noise level at -76 dB, i.e., few birds calling at a distance. **(C)** Audio spectrogram (left) and power spectrum (0.5 – 6 kHz, right) of background noise levels at -58 to -48 dB, i.e. many birds calling from short distance.

Table S1. Monthly specific acoustic presence of the anuran species at two wetlands in the VNP.

**Species**	**Malalo**						**Sandi**						
	IX	X	XI	XII	I	II	IX	X	XI	XII	I	II	III
Arthroleptis schubotzi	-	-	-	0.7%	-	-	-	-	-	-	-	-	-
Amietia desaegeri	-	-	-	-	-	-	-	2.0%	-	-	-	-	-
Hyperolius castaneus	29.2%	32.3%	45.8%	45.8%	72.2%	45.8%	66.2%	51.0%	47.5%	62.2%	97.2%	100%	100%
Hyperolius glandicolor	71.9%	53.1%	18.8%	47.9%	-	10.4%	-	-	35.8%	7.9%	-	-	-
Leptopelis karissimbensis	3.1%	5.0%	50%	37.5%	-	-	-	-	45.8%	7.4%	-	-	-
Leptopelis kivuensis	21.9%	10.4%	44.8%	50.0%	-	34.9%	-	-	44.2%	23.6%	-	-	-
Phrynobatrachus bequaerti	6.3%	41.7%	29.2%	33.3%	5.6%	21.9%	-	18.8%	49.2%	7.4%	15.3%	15.0%	-
